# Undergraduate dental curricula in Middle Eastern and Arabic-speaking African Nations − A cross-sectional study

**DOI:** 10.1016/j.sdentj.2024.10.003

**Published:** 2024-11-02

**Authors:** Lovely Muthiah Annamma, Jumma Al Khabuli, Sabrin Ali Azim, Huda Abutayyem, Mohamed Alkhuboli, A. Subaveerapandiyan, Rebecca Glanville, Kamran Ali

**Affiliations:** a Department of Clinical Sciences, Center of Medical and Bio-Allied Health Sciences Research, College of Dentistry, Ajman University, Ajman, United Arab Emirates; b Department of Preclinical Sciences, Oral Pathology, College of Dental Medicine, QU Health, Qatar University, Doha, Qatar; c Department of Oral Surgery, Gulf Medical University, College of Dentistry Ajman, United Arab Emirates; d Intern Tawam Hospital Alain, United Arab Emirates; e Librarian, Department of Library, Sai University, One Hub Road, Paiyanur, Chennai 603104, India; f Department of Library, Shinawatra University, Pathum Thani 12160, Thailand; g Assessment Psychometrician, Plymouth University, Faculty of Health, Plymouth PL4 8AA, UK; h Associate Dean Academic Affairs, College of Dental Medicine, QU Health, Qatar University, Doha, Qatar

**Keywords:** Curriculum, dentistry, Admission criteria, Arabic- speaking, Undergraduate

## Abstract

**Purpose:**

To evaluate the design of undergraduate curricula, teaching and learning practices, assessments, admission criteria and quality assurance in dental schools across Middle Eastern and Arab-speaking African nations.

**Methodology:**

A cross-sectional survey was sent to 40 dental colleges in the Middle East and African Arabic-speaking countries. A purposefully designed proforma consisting of 21 items divided into five sections based on admission criteria, curriculum delivery, teaching and learning practices, assessment methods, and quality assurance was used for data collection on Google forms. Participation in the survey was voluntary and all participants were consented before data collection. The data was analysed for descriptive statistics.

**Results:**

A total of 28 dental institutions participated in the study yielding a response rate of 70 %. The data showed variations in the admission criteria, curricula, and assessment methods. The reliability statistics were satisfactory (Cronbach’s alpha = 0.89). ANOVA showed significant differences were noted in the clinical experience of students by country and curriculum design (p ≤ 0.001). Gaps were also identified in the quality assurance processes at some of the participating institutions.

**Conclusion:**

This study provides a snapshot of undergraduate dental education in the Middle East and Arabic speaking African countries. Although a majority of the institutions follow a student-centered approach, some in institutions still follow the traditional teacher-centered approach which is not consistent with the contemporary strategies in healthcare education. The admission criteria are based on high school grades albeit with some variations. The duration of dental programs is five years at most institutions with or without an additional foundation year. Variations were also noted in the assessment weightings, clinical targets and quality assurance procedures. Further collaboration is required to facilitate harmonization of dental curricula in the region, solicit international recognition and to better support their graduates in pursuing postgraduate studies and employment options.

## 1 Introduction

The history of dentistry in the Arabian Peninsula can be traced back to the 10th century when seminal works by Muslim physicians Al-Razi, Ibne Sina and Ibne Al-Haitham laid the foundations of medicine and dentistry in Arab regions (Baqain et al., 2016). However, it took several centuries for the establishment of a dental institution and the first dental college offering a formal qualification in dentistry was established in the early 20th century. The last two decades have witnessed an exponential growth in the number of dental colleges in the Middle East (Baqain et al., 2016). Saudi Arabia has the highest number of dental colleges in the Middle East where the total number of college has grown to 26 with eight in the public sector and eight in the private sector (Alshihri et al., 2021). In addition, Qatar opened its first dental college in 2019 (Ali et al., 2022). Among Arabic-speaking African countries, Egypt has the highest number of dental colleges with 43 universities offering a Bachelor of Dental Surgery (BDS) program (Gouda et al., 2023).

While the growth of dental colleges in Arab countries is encouraging, in some countries the number of dental colleges exceeds national requirements. More importantly, there is a dearth of published studies on dental curricula, admission criteria, and compliance with academic standards in undergraduate dental education programs in the Middle East and Arabic-speaking African countries. Moreover, with increasing globalization, it is common for dental graduates to migrate to other countries, especially the West, to take advantage of opportunities for professional growth, financial stability, lifestyle choice and sometimes to evade the effects of regional conflicts (Balasubramanian et al., 2015, Balasubramanian et al., 2016, Balasubramanian et al., 2017, Balasubramanian and Short, 2011, Hajian et al., 2023).

Dental institutions in the Arab region need to ensure that the standards of dental education and training are on par with those in the developed countries and that their graduates are adequately prepared to serve their communities and capable of exploring their career options globally. This study aimed to analyze and evaluate the current admission criteria, curricular designs, teaching and learning practices, assessment methods, and quality assurance followed in Middle Eastern and Arabic-speaking African countries.

## 2 Methods

### 2.1 Research ethics

The study was carried out as per the Code of Ethics of the World Medical Association (Declaration of Helsinki) for experiments involving humans*.* Ethical approval for the study was obtained from the institutional research ethics committee (Application number D-H-F-11-Nov dated 20th December 2021). Participation in the study was voluntary and all data were processed anonymously. Informed consent for participation was provided by all participants.

### 2.2 Study design

A cross-sectional analytical study was employed in this study.

### 2.3 Settings

This study was carried out as a web-based online survey.

### 2.4 Study duration

The study was conducted in two phases. Phase 1 was used to pilot the study questionnaire for validation and was carried out from January 15 to February 2022. Phase 2 of the study was used for data collection based on the finalised questionnaire and was carried out from May 15 to July 25, 2022.

### 2.5 Data collection instrument

A questionnaire encompassing admission criteria, curriculum design, teaching and learning practices, assessments, and quality assurance for undergraduate dental programs was developed by the research team which consisted of experienced dental academics. The questionnaire was piloted with 12 faculty members at different institutions to determine the relevance, language and clarity of the questionnaire. Pearson’s correlation (r = 0.78) showed satisfactory correlations between continuous variables while Kendall’s Tau showed satisfactory correlations between ranked variables (τ = 0.76). Following the pilot, minor amendments were made to improve the language and clarity of four items and the survey questionnaire was finalized with consensus amongst the research team.

The definitive version of the survey questionnaire consisted of 21 questions divided into the following sections: 1) admission criteria, 2) curriculum design (title and duration of program, curriculum delivery), 3) teaching and learning practices; 4) assessment methods, and 5) quality assurance.

### 2.6 Sampling technique and participants

A Non probability purposive sampling technique was used to target representatives of eligible institutions, specifically institutions offering an undergraduate dental program. The Deans of target institutions were invited to participate in the study via institutional The invitations were accompanied by a participant information sheet and a questionnaire. Institutions interested in participating were asked to return the completed questionnaires by email to the corresponding researcher. An email reminder was sent after two weeks.

### 2.7 Data analysis

Descriptive statistics including confidence intervals were calculated for each item and for the combined dataset. Analysis of variance (ANOVA) was used to determine any significant variations between the results by country and curriculum type. Estimated marginal means were calculated from the ANOVA outcomes. All data were analyzed and visualized using RStudio (version 2023.06.2) incorporating R version 4.0.5.

## 3 Results

**Fig. 1:**
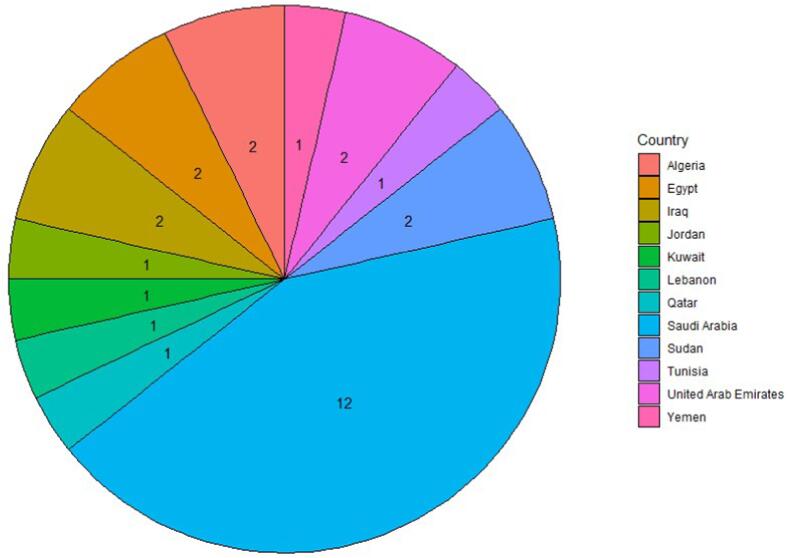
Geographic distribution of participating institutions.

From a total of 40 dental institutes invited to participate in the survey, 28 responses were received yielding a response rate of 70 %. The participating institutions were from Saudi Arabia, United Arab Emirates, Jordan, Iraq, Kuwait, Qatar, Lebanon, Yemen, Algeria, Egypt, Tunisia and Sudan. The geographic distribution of the participating institutions is depicted in Fig. 1. Details of individual institutions that participated in this study are provided in the supplementary file.

The data showed a combination of commonalities and differences in the undergraduate dental curricula, teaching and learning practices, assessment methods, and quality assurance procedures.

### 3.1 Admission criteria

The admission criteria require the applicants to have science subjects in high school. A minimum of 80 % marks are required by 82.6 % of institutions, 8.7 % of institutions require 75 % marks or more, and another 8.7 % require 65 % marks. In addition, 39.1 % institutions require applicants to have passed an English language test. Entrance exams are conducted by 30.4 % of institutions, while 47.8 % also require applicants to attend an interview. A practical test is required by only 14.28 % of institutions.

### 3.2 Curriculum design

#### 3.2.1 Title of the program

Regarding the titles of undergraduate dental programs at participating institutions, Bachelor of Dental Surgery (BDS) was the most common (82.6 %), followed by Doctor of Dental Surgery (DDS) and Doctor of Dental Medicine (DDM) in 4.3 % institutions each. English was reported as the official medium of instruction by a vast majority (95.%) of the participating institutions followed by French (4.3 %).

#### 3.2.2 Duration of the program

The duration of the undergraduate dental program, excluding the internship year ranges from six years (year 1 foundation plus five years) in 82.6 % of institutions to five years (year 1 foundation plus four years) in 4.3 % of institutions, while 13 % of institutions offer a five-year program without a foundation year. Only 4.3 % of institutions offer a four-year program without the need for a foundation year.

#### 3.2.3 Curriculum delivery

A traditional teacher-centered curriculum is followed at six institutions, while 10 institutions follow a student-centered problem-based learning (PBL) model. The remaining 12 institutions follow a hybrid curriculum. The academic calendar follows a semester system in 52.2 % of institutions while the remainder follow an annual system. Elective courses are offered at 66.7 % of the institutions.

### 3.3 Teaching and learning practices

#### 3.3.1 Learning environment

Although 73.3 % of institutions have a digital management system (LMS), a blended learning approach (face-to-face and online) is followed by 65.2 % of institutions while the remaining institutions only deliver the teaching and learning activities face-to-face. The learning objectives and presentations for the teaching sessions are always shared with the students in advance by 67.9 % of institutions, sometimes by 13.0 % and rarely by 4.3 % of institutions.

#### 3.3.2 Clinical experience

All institutions provide clinical experience to the students with minimum clinical targets for core dental procedures. The overall mean requirement for all items was 12.56 (95 % CI: 8.99 to 16.13). Descriptive values for each individual item can be found in Table 1.

**Table 1:** Descriptive values of minimum clinical targets (all respondents).

Domain	Mean	Standard deviation (±)	95 % CI (Lower)	95 % CI (Upper)
Bitewing radiographs	12.86	10.49	8.79	16.92
Periapical radiographs	11.79	9.83	7.97	15.6
Local anesthetic injections	25.36	8.38	22.11	28.61
Basic periodontal charting	11.79	6.7	9.19	14.38
Periodontics: Root surface debridement	13.21	8.19	10.04	16.39
Periodontics: Scaling and polishing	13.57	7.8	10.55	16.6
Caries removal	18.75	9.09	15.23	22.27
Temporary Fillings	15.54	4.78	13.68	17.39
Class I fillings	12.5	4.81	10.63	14.37
Class II fillings	11.07	4.16	9.46	12.69
Class III fillings	11.61	4.31	9.93	13.28
Class IV fillings	10.89	7.46	8	13.79
Class V fillings	11.79	6.7	9.19	14.38
Endodontics multirooted teeth	10.43	4.12	8.83	12.03
Endodontics single rooted teeth	12.68	6.16	10.29	15.07
Tooth Extractions	28.21	4.56	26.45	29.98
Ceramic crowns	10.18	5.85	7.91	12.45
Metal crowns	6.07	7.37	3.21	8.93
Pit and fissure sealants	11.07	8.64	7.72	14.42
Pulpotomy deciduous teeth	4.96	8.24	1.77	8.16
Stainless steel crowns	11.79	9.05	8.28	15.29
Apexification	0.29	1.01	−0.11	0.68
Overall	12.56	9.04	8.99	16.13

Descriptive statistics for clinical targets by curriculum type are summarised in Table 2.

**Table 2:** Descriptive values of clinical targets by curriculum type.

Domain	Mean			Standard Deviation (±)			95 % CI (Lower)			95 % CI (Upper)		
	**Hybrid**	**PBL**	**Trad**	**Hybrid**	**PBL**	**Trad**	**Hybrid**	**PBL**	**Trad**	**Hybrid**	**PBL**	**Trad**
Bitewing radiographs	13.33	14	10	10.73	10.75	10.95	6.98	7.03	0.82	19.69	20.97	19.18
Periapical radiographs	11.67	15	6.67	9.37	11.79	5.16	6.11	7.35	2.34	17.22	22.65	10.99
Local anesthetic injections	29.17	23	21.67	2.89	9.49	11.69	27.46	16.84	11.87	30.88	29.16	31.46
Basic periodontal charting	10	14	11.67	0	8.43	9.83	10	8.53	3.43	10	19.47	19.9
Periodontics: Root surface debridement	12.5	17	8.33	6.22	9.49	7.53	8.82	10.84	2.03	16.18	23.16	14.64
Periodontics: Scaling and polishing	11.67	18	10	5.77	10.33	0	8.25	11.3	10	15.09	24.7	10
Caries removal	20	20.5	13.33	7.69	8.32	12.11	15.45	15.1	3.19	24.55	25.9	23.48
Temporary fillings	14.58	16	16.67	5.42	5.16	2.58	11.37	12.65	14.5	17.79	19.35	18.83
Class I fillings	12.08	14	10.83	3.96	6.58	2.04	9.73	9.73	9.12	14.43	18.27	12.54
Class II fillings	10	13	10	0	6.75	0	10	8.62	10	10	17.38	10
Class III fillings	10.83	13.5	10	1.95	6.69	0	9.68	9.16	10	11.99	17.84	10
Class IV fillings	10.83	11	10.83	6.69	8.76	8.01	6.87	5.32	4.12	14.79	16.68	17.54
Class V fillings	9.17	14	13.33	2.89	6.99	10.33	7.46	9.46	4.68	10.88	18.54	21.98
Endodontics multirooted teeth	10	10.2	11.67	0	6.36	4.08	10	6.08	8.25	10	14.32	15.09
Endodontics single rooted teeth	10.83	15.5	11.67	2.89	8.96	4.08	9.12	9.69	8.25	12.54	21.31	15.09
Tooth extractions	27.92	28.5	28.33	4.98	4.74	4.08	24.97	25.42	24.91	30.87	31.58	31.75
Metal crowns	4.17	7	8.33	5.15	10.59	4.08	1.12	0.13	4.91	7.22	13.87	11.75
Ceramic crowns	10	11.5	8.33	4.26	8.18	4.08	7.47	6.19	4.91	12.53	16.81	11.75
Pit and fissure sealants	9.58	13.5	10	6.2	10.01	10.95	5.91	7	0.82	13.26	20	19.18
Pulpotomy deciduous teeth	2.92	4	10.67	4.5	9.66	10.17	0.25	−2.27	2.15	5.58	10.27	19.19
Stainless steel crown	8.33	14	15	3.89	10.75	12.25	6.03	7.03	4.74	10.64	20.97	25.26
Apexification	0	0.7	0.17	0	1.64	0.41	0	−0.36	−0.18	0	1.76	0.51
Overall	11.80	13.99	11.70	8.28	9.94	8.68	10.93	12.95	10.79	12.67	15.04	12.62

The reliability statistics for student clinical experience at the participating institution (n = 28) were satisfactory as shown in Table 3.

**Table 3:** Reliability statistics.

Statistic	Value
Number of participating institutions	28
Cronbach's Alpha	0.891
Variance (%) due to institution	16.22
Variance (%) due to domain	39.94
Variance (%) residual	43.84
G coefficient	0.425
Relative SEM	428.11
Phi coefficient	0.38
Absolute coefficient	591.81

Analysis of variance identified significant variation by Country, Curriculum and Domain as summarised in Table 4. Institutions using a hybrid model had the lowest targets and those following a student-centered curriculum set the highest targets.

**Table 4:** Analysis of variance (all domains).

Factor	Df	Sum of Sq	RSS	AIC	F-statistic	P-value
Country	11	3124.122	29124.2	2423.344	6.347	<0.001
Curriculum Design	2	654.604	26654.68	2386.763	7.314	0.001
Domain	21	20404.64	46404.71	2690.296	21.713	<0.001

### 3.4 Assessments

A fixed pass mark is used for summative assessments by 56.5 % of institutions while 43.5 % of institutions employ standard setting methods for determining the pass mark for individual assessments. Only a few participating institutions assess pre-clinical skills of the undergraduate students in simulated dental learning environments. These include preclinical skills in operative dentistry and prosthodontics (22.4 % of institutions) followed by endodontics (21.4 % of institutions) dental radiology (17.3 % of institutions), and periodontics (11.2 % of institutions) while oral surgery skills are assessed at only 6.1 % of institutions.

The relative weightings of summative and formative assessments in preclinical and clinical courses at the participating institutions are summarised in Table 5.

**Table 5:** Weightings of pre-clinical and clinical courses.

Type of Courses	Weighting	Percentage (%)
*Preclinical courses*		
	80 % summative (final exams) and 20 % formative assessments	8.7
	60 % summative (final exams) and 40 % formative assessments	43.5
	50 % summative (final exams) and 50 % formative assessments	30.4
	Others	17.4

*Clinical courses*		
	60 % summative (final exams) and 40 % formative assessments	53.3
	50 % summative (final exams) and 50 % formative assessments	33.3
	Others	13.3

### 3.5 Quality assurance

All participating institutions have a quality assurance committee structure. The frequency of meetings of the quality assurance committee varies from every three months (43.5 %); every six months (30.4 %), or annually (26.1 %). However, 5.4 % of institutions reported that it took more than one year to convene a meeting of the relevant quality assurance committee. Student satisfaction surveys are conducted annually by 78.3 % of institutions and once every two years by 17.4 % of institutions. However, 4.3 % of institutions do not conduct any student satisfaction surveys. Regarding the assessment standards, 53.3 % benchmark their undergraduate assessments against the standards set by the national registration body, while 33.3 % benchmark them against the assessment standards used by other institutions. Benchmarking of assessment standards is undertaken by 13.3 % of institutions. Finally, a qualification in medical education is an essential requirement for 50 % of the participating institutions, desirable by 37.5 %, and not required for the remaining institutions.

## 4 Discussion

Given the wide variations in undergraduate dental curricula, it is difficult to identify a standard curriculum. Nevertheless, contemporary undergraduate dental curricula have a common goal: to equip dental students with the necessary scientific knowledge, clinical skills and affective attributes to prepare them for delivering safe, and evidence-based dental services to the community after graduation (Ali et al., 2014). Dental institutions across the globe share this common goal and undergraduate dental educati1on is most commonly delivered in university settings as a structured dental program ranging from four to six years. The variations in the duration of dental programs are partly related to the requirements of a foundation year. Dental programs at a vast majority of the institutions (n = 23) are structured over five years (year I foundation plus four years), while 3 institutions offer a five-year program without a foundation year. One institution offers a six-year program in which the internship year after graduation is included. Only one institution offers a four-year program without the need for a foundation year.

Contemporary education standards in dentistry follow a competency-based model with a student-centered approach to develop them as independent, reflective, and life-long learners and train them to uphold the highest standards of professionalism and commitment to the promotion of oral and dental health (Tonni et al., 2020). While undergraduate dental curricula can show variations in structure and design, it is expected that all dental programs will provide education in basic medical and dental sciences and provide training in core skills in preclinical and clinical settings. Moreover, dental students should be supported in developing their skills in communication, teamwork, management, leadership and professionalism. This study explored multiple dimensions of undergraduate dental education in Middle Eastern and Arabic-speaking African countries and evaluated the curriculum design, teaching and learning methods, assessments, quality assurance and admission criteria in the target institutions.

Responses were received from dental institutions in the Middle East including Saudi Arabia, the United Arab Emirates, Kuwait, Jordan, Iraq, Lebanon, and Qatar as well as Arabic-speaking African countries including Egypt, Algeria, Tunisia, and Sudan. Only a small percentage of the responses to the survey were provided by the deans (7.1 %) and the majority of responses were provided by their nominated dental faculty representatives, who are members of the curriculum committee. Overall, the curriculum structure, teaching and learning methods, assessments, and quality assurance processes are comparable to global trends in undergraduate dental education albeit with some deficiencies in a small percentage of participating institutions. Given that the majority of the institutions have English as the official medium of instruction, along with French, this enables graduates from these institutions to pursue postgraduate qualifications and license examinations in English/French-speaking countries in the United States, Canada, the United Kingdom, Australia and some European countries without facing any language barriers (Bissell et al., 2016).

The admission criteria for undergraduate dental programs are largely similar to those reported in other countries globally. However, candidate interviews were only reported to be a requirement in only 42 % of the institutions. In many Western countries, Interviews are a desirable component of admission criteria and may allow a more objective evaluation of the communication skills, motivation, readiness for the profession, service, and problem-solving abilities of prospective candidates (Duff et al., 2020, Glazer et al., 2016). Nevertheless, it is recognized that conducting interviews with prospective candidates requires a considerable resource, especially for institutions with a large intake and not all dental institutions may be able to reserve such resources routinely. There is growing evidence to support the use of practical tests for admissions to undergraduate dental programs (Abd Alraheam et al., 2022, Arnold et al., 2011, Beier et al., 2010, Kothe et al., 2014). However, as observed in the present study, only a few institutions use practical tests for dental admissions. In the light of the best evidence, There is a need to standardize practical tests as a filter for dental admissions and it is conceivable that the trend of practical tests for dental admissions may grow further in the future.

The teaching and learning practices reported by the participating institutions are largely similar to those reported by dental institutions globally (Ali et al., 2016a). Nevertheless, curriculum development is a dynamic process and needs a continuous review to meet the oral health needs of communities in the 21st century (McHarg and Kay, 2008, Perry et al., 2017). A fundamental goal of undergraduate dental education should be to bridge the gap between the basic sciences and clinical disciplines through a meaningful vertical integration to achieve the transfer of basic sciences knowledge into clinical practice (Ali et al., 2020). Technological developments are happening at a lightning pace and the growing use of digital dentistry, immersive realities, and artificial intelligence warrant a careful rethinking of how dental education should be adapted to prepare dental graduates for the next 40 years (Ali et al., 2024, Ba-Hattab et al., 2023, Daud et al., 2023, Kim et al., 2023, Monterubbianesi et al., 2022, Philip et al., 2023).

Significant variations were observed in the requirements for clinical targets (p ≤ 0.001). Nevertheless, the scope of clinical targets and experiences for undergraduate dental students in the present study appears to fulfill the core requirements of a contemporary dental program (Ali et al., 2017a, Ali et al., 2017b, Mat Yudin et al., 2020) Notwithstanding the differences in the number of clinical targets, it is clear that competence in these core dental skills would allow dental students to be adequately prepared for their transition into independent clinical practice (Ali et al., 2016b).

Variations were also observed in the relative weightings of summative and formative assessments among the participating institutions with weightings of summative assessments accounting for 50–80 % to make decisions regarding student progression. Although there is no gold standard for the relative weightings of summative and formative assessments, it is important that decisions regarding student progression are based on assessments that conform to contemporary standards of assessments including the use of multiple assessment strategies and frequent assessments throughout the academic year rather than being limited to end of year assessments (Lockyer et al., 2017).

The results show that all participating institutions have an existing committee structure to oversee the quality assurance of undergraduate dental programs. However, some apparent deficiencies in quality assurance were identified. Firstly, the frequency of meetings of the quality assurance committee was reported to be once per year or even more by some institutions, which raises some concerns. Quality assurance is a fundamental tool to ensure that contemporary educational and assessment standards are maintained in an educational program and that graduates are qualified to provide safe healthcare services to communities (Busari, 2012, Sjöström et al., 2019). Moreover, robust quality assurance mechanisms also enable institutions to prepare for international accreditation (Yoshioka & Nara, 2013). It is suggested that institutions with infrequent meetings of relevant quality assurance committees revisit their existing processes and enhance this crucial aspect of dental education to improve the learning experiences of their students. Similarly, some institutions reported that they do not conduct any student surveys at the end of each academic year (Langan and Harris, 2023, Rigopoulos, 2022, Williams and Kane, 2008). Students are undoubtedly the biggest stakeholders in an educational program, and their feedback serves to inform future curriculum development and improvements in teaching and learning practices as well to enhance the quality and transparency of the assessments. Finally, formal qualifications in medical education have been shown to enhance the agency of educators in the healthcare professions (Du et al., 2022). Many medical and dental institutions in the West require all teaching faculty to have formal certification in medical education (Eitel et al., 2000, Fellow-Smith et al., 2013, Görlitz et al., 2015, Schiekirka-Schwake et al., 2017). It is recommended that this be considered a desirable, if not an essential attribute for all dental faculty members. Quality assurance of dental education requires a multi-pronged strategy to evaluate all dimensions of the curriculum, teaching and learning practices, and assessment methods and the afore-mentioned recommendations indicate only some of the measures that may contribute to effective quality assurance.

The dental program structure, duration and admission criteria in the participating institutions are largely similar to those in the United Kingdom, Europe, and Asia (Chuenjitwongsa et al., 2018, Council, 2015). However, an increasing number of dental institutions in the West require interviews as part of the admission process (Arnold et al., 2011, Beier et al., 2010, Glazer et al., 2016). At the same time, dental schools in the USA and Canada offer a four-year dental program but applicants require a bachelor’s science degree as part of the admission criteria. The Association for Dental Education in Europe (ADEE) has been working to harmonize undergraduate dental education across Europe and has developed the profile and competencies expected of a European dental graduate (Cowpe et al., 2010, Field et al., 2017). The aim is to develop uniform standards of undergraduate dental education and allow free movement of dentists across Europe without the need for additional license examinations. Dental regulatory bodies in the Middle East may follow a similar approach and develop a common framework for dental education. It is recognised that it may not be possible to bridge all the variations observed in undergraduate dental education, the initial steps would be to develop consensus on the learning outcomes of undergraduate dental education as achieved by ADEE and to develop uniformity in how credit hours for undergraduate dental education are calculated (Cowpe et al., 2010, Kirnbauer and Ali, 2020). Moreover, the dental institutions in the Middle East can work together to develop and implement a common framework for quality assurance of their dental education programs. A similar approach could be followed by dental institutions in Africa.

This study has some limitations. Although responses were received from the majority of the institutions invited to participate in the study, only a small fraction of dental programs in the Middle East and Arabic-speaking African countries were represented in the study. Therefore, the findings may be best regarded as preliminary and should be interpreted with a degree of caution. Second, the findings are based on closed-ended questions and future research involving qualitative methods that may provide a more in-depth understanding of various dimensions of dental curricula and educational strategies at participating institutions along with associated challenges. Furthermore, longitudinal studies may provide more dynamic insights into trends in the evolution of dental curricula. More importantly, not all dimensions of curricula, teaching and learning methods, and assessments could be explored in a single survey. Further studies with focused questions on specific elements of curriculum design, learning methods and assessments are required for a more comprehensive evaluation. The mapping and alignment of teaching, learning and assessment methods to program learning outcomes is a key requirement for programmatic assessment in contemporary healthcare education. Therefore, it is recommended that future studies explore this dimension (Heeneman et al., 2021). The assimilation and impact of technology in dental education is another topical issue in contemporary dental education and that needs to be explored with focused studies on the incorporation of modern technologies such as virtual reality with haptic feedback, digital dentistry, and artificial intelligence. Nevertheless, this study is a first step in evaluation and harmonization of dental education in Arabic speaking countries across the Middle East and African region and would provide impetus for further collaboration among the participating institutions.

## 5 Conclusion

This study provides a snapshot of undergraduate dental education in Middle Eastern and Arabic-speaking African countries. Although a majority of the institutions follow a student-centered approach in the delivery of dental curricula, some institutions still follow the traditional teacher-centered model, which is not consistent with contemporary strategies in healthcare education. The admission criteria for dental programs are based on high school grades albeit with some variations. The duration of dental programs at most institutions is five years, with or without an additional foundation year. Variations were also noted in the assessment weightings, clinical targets, and quality assurance procedures. Further collaboration is required to facilitate the harmonization of dental curricula in the region, solicit international recognition, and better support their graduates in pursuing postgraduate studies and employment options.

## 6 Research ethics

Ethics approval for the study was obtained from the institutional research ethics committee. Application number D-H-F-11-Nov dated 20th December 2021). Participation in the study was voluntary and all data was processed anonymously.

## 7 Declaration of generative AI in scientific writing

Generative AI was not used for any aspect of this study including drafting of the manuscript.

## Authors contributions

KA conceptualized the study, developed the methodology and drafted the manuscript; LMA, JK, SAA, HA, MA and SA contributed to data collection; RG contributed to data analysis; all authors reviewed and approved the manuscript.

## Funding

Open access funding for this study was provided by the Journal Unit, King Saud University, Saudi Arabia.

## Declaration of competing interest

The authors declare that they have no known competing financial interests or personal relationships that could have appeared to influence the work reported in this paper.
