# Supplementary material for: Undergraduate dental curricula in Middle Eastern and Arabic-speaking African Nations − A cross-sectional study
Source: Saudi Dent J. 2024 Nov 2;36(12):1681–7. doi: 10.1016/j.sdentj.2024.10.003 (PMC11976074; doi:10.1016/j.sdentj.2024.10.003)
Supplement: Supplementary Data 1 [file mmc1.pdf]

Dental Curricula

By Kamran Ali

WORD COUNT	6443	TIME SUBMITTED	18-SEP-2024 07:27PM
		PAPER ID	111791399

Undergraduate Dental Curricula in Middle Eastern and Arabic-speaking African Nations --- A Cross-sectional Study.

Abstract

Purpose: To evaluate the design of undergraduate curricula, teaching and learning practices, assessments, admission criteria and quality assurance in dental schools across Middle Eastern and Arab-speaking African nations

Methodology: A cross-sectional survey was sent to 40 dental colleges in the Middle East and African Arabic-speaking countries. A purposefully designed proforma consisting of 21 items divided into five sections based on admission criteria, curriculum delivery, teaching and learning practices, assessment methods, and quality assurance was used for data collection on Google forms. The Deans of dental colleges were invited to complete the survey questionnaire online. Participation in the survey was voluntary and all participants were consented before data collection. The data was analysed for descriptive statistics.

Results: A total of 28 dental institutions participated in the study yielding a response rate of 70%. The data showed variations in the admission criteria, curricula, and assessment methods. The reliability statistics were satisfactory (Cronbach's alpha= 0.89). ANOVA showed significant differences were noted in the clinical experience of students by country and curriculum design ($p \leq 0.001$). Gaps were also identified in the quality assurance processes at some of the participating institutions.

Conclusion: This study provides a snapshot of undergraduate dental education in the Middle East and Arabic speaking African countries. Although a majority of the institutions follow a student-centered approach in the delivery of dental curricula, some in institutions still follow the traditional teacher-centered which is not consistent with the contemporary strategies in healthcare education. The admission criteria to the dental programmes are based on high school grades albeit with some variations. The duration of dental programmes is five years at most institutions with or without an additional foundation year. Variations were also noted in the assessment weightings, clinical targets and quality assurance procedures. Further collaboration is required to facilitate harmonization of dental curricula in the region, solicit international recognition and to better support their graduates in pursuing postgraduate studies and employment options.

Keywords *curriculum, dentistry; admission criteria; Arabic- speaking; undergraduate*

1 Introduction

The history of dentistry in the Arabian peninsula can be traced back to the 10th century when seminal works by Muslim physicians Al-Razi, Ibne Sina and Ibne Al-Haitham laid the foundations of medicine and dentistry in the Arab region.(Baqain et al., 2016) However, it took several centuries for the establishment of a dental institution and the first dental college offering a formal qualification in Dentistry was established in the early 20th century. The last two decades have witnessed an exponential growth in the number of dental colleges in the Middle East.(Baqain et al., 2016) Saudi Arabia has the highest number of dental colleges in the Middle East where the total number of college has grown to 26 with 8 in the public sectors and 8 in the private sector.(Alshihri et al., 2021) In addition, Qatar opened its first dental college in 2019.(Ali et al., 2022) Amongst the Arabic-speaking African countries, Egypt has the highest number of dental colleges with 43 universities offering a Bachelor of Dental Surgery (BDS) program.(Gouda et al., 2023)

While the growth of dental colleges in the Arab countries is encouraging, the number of dental colleges exceeds the national requirements in some countries. More importantly, is a dearth of published studies on dental curricula, admission criteria, and compliance with academic standards in undergraduate dental education programmes in the Middle East and Arabic speaking African countries. Moreover, with increasing globalization, it is common for dental graduates to migrate to other countries, especially the West, to avail opportunities for professional growth, financial stability, lifestyle choice and sometimes to evade the effects of regional conflicts.(Balasubramanian et al., 2015, 2016, 2017; Balasubramanian & Short, 2011; Hajian et al., 2023)

Dental institutions in the Arab region need to ensure that the standards of dental education and training are at par with those in the developed countries and their graduates are adequately prepared to serve their communities and also capable of exploring their career options globally. This study aimed to analyze and evaluate the current admission criteria, curricular designs, teaching and learning practices, assessment methods, and quality assurance followed in Middle Eastern and Arabic-speaking African countries.

2 Methods

2.1 Research ethics

The study was carried out as per The Code of Ethics of the World Medical Association (Declaration of Helsinki) for experiments involving humans. Ethics approval for the study was obtained from the institutional research ethics committee (Application number D-H-F-11-Nov dated 20th December 2021). Participation in the study was voluntary and all data were processed anonymously. Informed consent for participation was provided by all participants.

2.2 Study design

A cross-sectional analytical study was employed for this study.

2.3 Settings:

This study was carried out as a web-based online survey

2.4 Study duration

The study was conducted in two phases. Phase 1 was used to pilot the study questionnaire for validation and was carried out from 15 January to 14 February 2022. Phase 2 of the study was used for data collection based on the finalised questionnaire and was carried out from 15th May to 25th July 2022.

2.5 Data collection instrument

A questionnaire encompassing admission criteria, curriculum design, teaching and learning practices, assessments, and quality assurance for undergraduate dental programmes was developed by the research team consisting of experienced dental academics. The questionnaire was piloted with 12 faculty members at different institutions to determine the relevance, language and clarity of the questionnaire. Pearson correlation (r=0.78) showed satisfactory correlations between continuous variables while Kendall's Tau showed satisfactory correlations between ranked variables (τ=0.76). were used to Following the pilot, minor amendments were made to improve the language and clarity of 4 items and the survey questionnaire was finalised with consensus amongst the research team.

The final version of the survey questionnaire consisted of 21 questions divided into the following section: 1) admission criteria; 2) curriculum design (title and duration of program, curriculum delivery); 3) teaching and learning practices; 4) assessment methods; and 5) quality assurance.

2.6 Sampling technique and participants

Non probability purposive sampling technique was used to target representatives of eligible institutions i.e., institutions offering an undergraduate dental program. The Deans of target institutions were invited to participate in the study via institutional email. The invites were accompanied by a participant information sheet and the questionnaire. Institutions who were interested in participating were asked to return the completed questionnaires by email to the corresponding researcher. An email reminder was sent after two weeks.

2.7 Data analysis

Descriptive statistics including confidence intervals were calculated for each item and for the combined dataset. Analysis of Variance was used to determine any significant variation between the results by country and curriculum type. Estimated marginal means were calculated from the ANOVA outcomes. All data were analysed and visualized using RStudio (version 2023.06.2) incorporating R version 4.0.5.

3 Results

From a total of 40 dental institutes invited to participate in the survey, 28 responses were received yielding a response rate of 70%. The participating institutions were from Saudi Arabia, United Arab Emirates, Jordan, Iraq, Kuwait, Qatar, Lebanon, Yemen, Algeria, Egypt, Tunisia and Sudan. The geographic distribution of participating institutions is depicted in Figure 1. Details of individual institutions which participated in this study are provided in the supplementary file.

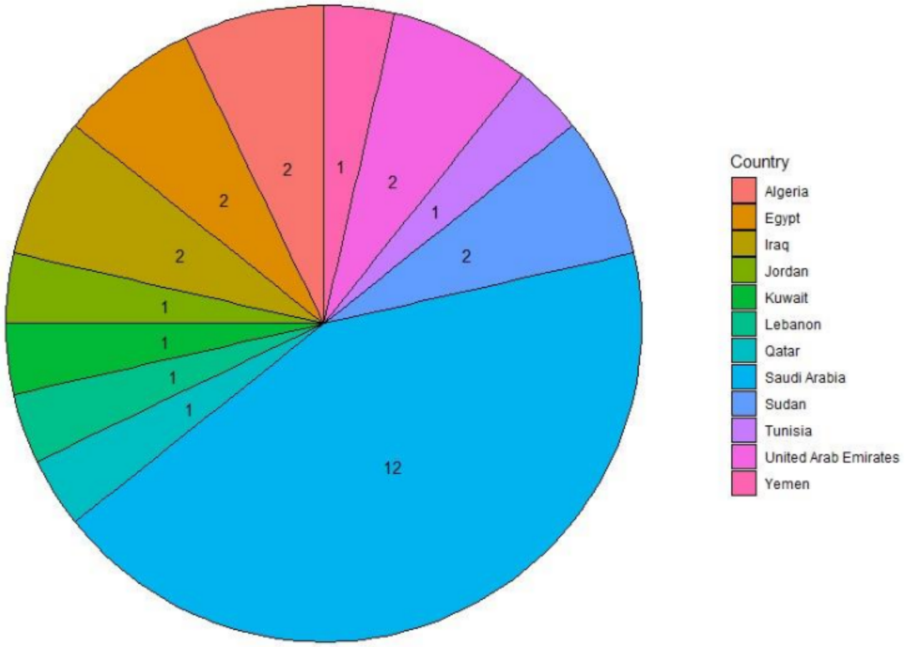

Figure 1: Geographic distribution of participating institutions.

The data showed a combination of commonalities and differences in the undergraduate dental curricula, teaching and learning practices, assessment methods, and quality assurance procedures.

3.1 Admission criteria

The admission criteria require the applicants to have science subjects in high school. A minimum of 80% marks are required by 82.6% institutions, while 8.7% institutions require 75% marks or more, and another 8.7% require 65% marks. In addition, 39.1% institutions also

require the applicants to have passed an English language. Entrance exams are conducted by 30.4% of institutions, while 47.8% also require the applicants to attend an interview. A practical test is required by only 14.28% of institutions.

3.2 Curriculum design

3.2.1. Title of the program

Regarding the titles of undergraduate dental programs at participating institutions, Bachelor of Dental Surgery (BDS) was the most common title (82.6%) followed by Doctor of Dental Surgery (DDS) and Doctor of Dental Medicine (DDM) in 4.3% institutions each. English was reported as the official medium of instruction by a vast majority (95.%) of participating institutions followed by French (4.3%).

3.2.2 Duration of the programme

The duration of the undergraduate dental programme, excluding the internship year ranges from 6 years (I year foundation plus 5 years) in 82.6% of institutions; 5 years (I year foundation plus 4 years) in 4.3% institutions; while 13% institutions offer a five-year program without a foundation year. Only 4.3% of institutions offer a four-year program without the need for a foundation year.

3.2.3 Curriculum delivery

A traditionall teacher centred curriculum is followed at six institutions, while 10 institutions follow a student-centred problem-based learning (PBL) model. The remaining 12 institutions follow a hybrid curriculum. The academic calender follows a semester system in 52.2% institutions while the remaining follow an annual system. Elective courses are offered at 66.7% of institutions.

3.3 Teaching and Learning Practices

3.3.1 Learning environment

Although 73.3% institutions have a digital management system (LMS), a blended learning approach (face-to-face and online) is followed by 65.2% institutions while the remaining institutions only deliver the teaching and learning activities face-to-face only. The learning objectives and presentations for the teaching sessions are always shared with the students in

advance by 67.9% institutions, sometimes by 13.0 % and rarely by 4.3% institutions.

3.3.2 Clinical Experience

All institutions provide clinical experience to the students with minimum clinical targets for core dental procedures. The overall mean requirement for all items was 12.56 (95%CI 8.99 to 16.13). Descriptive values for each individual item can be found in Table 1.

Table 1: Descriptive Values of Minimum Clinical Targets (all respondents)

**Domain**	**Mean**	**Standard Deviation (±)**	**95% CI (Lower)**	**95% CI (Upper)**
Bitewing radiographs	12.86	10.49	8.79	16.92
Periapical radiographs	11.79	9.83	7.97	15.6
Local anesthetic injections	25.36	8.38	22.11	28.61
Basic periodontal charting	11.79	6.7	9.19	14.38
Periodontics: Root surface debridement	13.21	8.19	10.04	16.39
Periodontics: Scaling and polishing	13.57	7.8	10.55	16.6
Caries removal	18.75	9.09	15.23	22.27
Temporary Fillings	15.54	4.78	13.68	17.39
Class I fillings	12.5	4.81	10.63	14.37
Class II fillings	11.07	4.16	9.46	12.69
Class III fillings	11.61	4.31	9.93	13.28
Class IV fillings	10.89	7.46	8	13.79
Class V fillings	11.79	6.7	9.19	14.38
Endodontics multirooted teeth	10.43	4.12	8.83	12.03
Endodontics single rooted teeth	12.68	6.16	10.29	15.07
Tooth Extractions	28.21	4.56	26.45	29.98
Ceramic crowns	10.18	5.85	7.91	12.45
Metal crowns	6.07	7.37	3.21	8.93
Pit and fissure sealants	11.07	8.64	7.72	14.42
Pulpotomy deciduous teeth	4.96	8.24	1.77	8.16
Stainless steel crowns	11.79	9.05	8.28	15.29
Apexification	0.29	1.01	-0.11	0.68
Overall	12.56	9.04	8.99	16.13

Descriptive statistics for clinical targets by curriculum type are summarised in Table 2.

Table 2: Descriptive values of Clinical Targets by Curriculum Type

Domain	Mean			Standard Deviation (±)			95% CI (Lower)			95% CI (Upper)		
	Hybrid	PBL	Trad	Hybrid	PBL	Trad	Hybrid	PBL	Trad	Hybrid	PBL	Trad
Bitewing radiographs	13.33	14	10	10.73	10.75	10.95	6.98	7.03	0.82	19.69	20.97	19.18
Periapical radiographs	11.67	15	6.67	9.37	11.79	5.16	6.11	7.35	2.34	17.22	22.65	10.99
Local anesthetic injections	29.17	23	21.67	2.89	9.49	11.69	27.46	16.84	11.87	30.88	29.16	31.46
Basic periodontal charting	10	14	11.67	0	8.43	9.83	10	8.53	3.43	10	19.47	19.9
Periodontics: Root surface debridement	12.5	17	8.33	6.22	9.49	7.53	8.82	10.84	2.03	16.18	23.16	14.64
Periodontics: Scaling and polishing	11.67	18	10	5.77	10.33	0	8.25	11.3	10	15.09	24.7	10
Caries removal	20	20.5	13.33	7.69	8.32	12.11	15.45	15.1	3.19	24.55	25.9	23.48
Temporary fillings	14.58	16	16.67	5.42	5.16	2.58	11.37	12.65	14.5	17.79	19.35	18.83
Class I fillings	12.08	14	10.83	3.96	6.58	2.04	9.73	9.73	9.12	14.43	18.27	12.54
Class II fillings	10	13	10	0	6.75	0	10	8.62	10	10	17.38	10
Class III fillings	10.83	13.5	10	1.95	6.69	0	9.68	9.16	10	11.99	17.84	10
Class IV fillings	10.83	11	10.83	6.69	8.76	8.01	6.87	5.32	4.12	14.79	16.68	17.54
Class V fillings	9.17	14	13.33	2.89	6.99	10.33	7.46	9.46	4.68	10.88	18.54	21.98
Endodontics multirooted teeth	10	10.2	11.67	0	6.36	4.08	10	6.08	8.25	10	14.32	15.09
Endodontics single rooted teeth	10.83	15.5	11.67	2.89	8.96	4.08	9.12	9.69	8.25	12.54	21.31	15.09
Tooth extractions	27.92	28.5	28.33	4.98	4.74	4.08	24.97	25.42	24.91	30.87	31.58	31.75
Metal crowns	4.17	7	8.33	5.15	10.59	4.08	1.12	0.13	4.91	7.22	13.87	11.75
Ceramic crowns	10	11.5	8.33	4.26	8.18	4.08	7.47	6.19	4.91	12.53	16.81	11.75
Pit and fissure sealants	9.58	13.5	10	6.2	10.01	10.95	5.91	7	0.82	13.26	20	19.18
Pulpotomy deciduous teeth	2.92	4	10.67	4.5	9.66	10.17	0.25	-2.27	2.15	5.58	10.27	19.19
Stainless steel crown	8.33	14	15	3.89	10.75	12.25	6.03	7.03	4.74	10.64	20.97	25.26
Apexification	0	0.7	0.17	0	1.64	0.41	0	-0.36	-0.18	0	1.76	0.51
Overall	11.80	13.99	11.70	8.28	9.94	8.68	10.93	12.95	10.79	12.67	15.04	12.62

The reliability statistics for student clinical experience at the participating institution (n=28) were satisfactory as shown in Table 3.

Table 3: Reliability statistics

Statistic	Value
N Participating institutions	28
Cronbach's Alpha	0.891
Variance (%) due to institution	16.22
Variance (%) due to domain	39.94
Variance (%) residual	43.84
G coefficient	0.425
Relative SEM	428.11
Phi coefficient	0.38
Absolute coefficient	591.81

Analysis of variance identified significant variation by Country, Curriculum and Domain as summarised in Table 4. Institutions using a hybrid model had the lowest targets and those following a student-centered curriculum set the highest targets.

Table 4: Analysis of Variance (all domains)

Factor	Df	Sum of Sq	RSS	AIC	F-statistic	P-value
Country	11	3124.122	29124.2	2423.344	6.347	<0.001
Curriculum Design	2	654.604	26654.68	2386.763	7.314	0.001
Domain	21	20404.64	46404.71	2690.296	21.713	<0.001

3.4 Assessments

A fixed pass mark is used for summative assessments by 56.5% institutions while 43.5% institutions employ standard setting methods for determining the pass mark for individual assessments. Only a few participating institutions assess pre-clinical skills of the undergraduate students in simulated dental learning environments. These include preclinical skills in operative dentistry and prosthodontics (22.4% institutions) followed by endodontics (21.4% institutions) dental radiology (17.3% institutions), and periodontics (11.2% institutions) while oral surgery skills are assessed at only 6.1% institutions.

The relative weightings of summative and formative assessments in preclinical and clinical courses at the participating institutions are summarised in Table 5.

Table 5: Weightings of Pre-clinical and Clinical Courses

Type of Courses	Weighting	Percentage (%)
Preclinical courses		
	80% summative (final exams) and 20% formative assessments	8.7
	60% summative (final exams) and 40% formative assessments	43.5
	50% summative (final exams) and 50% formative assessments	30.4
	Others	17.4
Clinical courses		
	60% summative (final exams) and 40% formative assessments	53.3
	50% summative (final exams) and 50% formative assessments	33.3
	Others	13.3

3.5 Quality assurance

All participating institutions have a quality assurance committee structure. The frequency of meetings of the quality assurance committee varies from 3-monthly (43.5%); 6-monthly (30.4%) or annually (26.1%). However, 5.4% of institutions reported that it took more than one year to convene a meeting of the relevant quality assurance committee. Student satisfaction surveys are conducted annually by 78.3% of institutions and once every two years by 17.4% institutions. However, 4.3% of institutions do not conduct any student satisfaction surveys. Regarding the assessment standards, 53.3% benchmark their undergraduate assessments against the standards set by the national registration body while 33.3% benchmark it against assessment standards used by other institutions. Benchmarking of assessment standards is undertaken by 13.3% of institutions. Finally, a qualification in medical education is an essential requirement for 50% of participating institutions, desirable by 37.5 % and not required for the remaining institutions.

4 Discussion:

Given the large variations in undergraduate dental curricula, it is difficult to identify a standard curriculum. Nevertheless, contemporary undergraduate dental curricula have a common goal i.e., to equip the dental students with scientific knowledge, clinical skills and affective attributes to prepare them for delivering safe and evidence-based dental services to the community after graduation.(Ali et al., 2014) Dental institutions across the globe share this common goal and undergraduate dental education is most commonly delivered in university settings as a structured dental programme ranging from 4-6 years. The variations in the duration of the dental programmes are partly related to the requirement of a foundation year. Dental programmes at a vast majority of the institutions (n=23) are structured over five years (I year foundation plus 4 years) ; while 3 institutions offer a five-year program without a foundation year. One institution offers a six-year programme whereby the internship year after graduation is included in the programme. Only one institution offers a four-year program without the need for a foundation year.

Contemporary education standards in dentistry follow a competency-based model with a student-centered approach to develop them as independent, reflective, and life-long learners and train them to uphold the highest standards of professionalism and commitment to the promotion of oral and dental health.(Tonni et al., 2020) Whilst undergraduate dental curricula can show variations in structure and design, it is expected that all dental programmes provide education in basic medical and dental sciences, provide training in core skills in preclinical and clinical settings. Moreover, dental students should be supported to develop their skills in communication, teamworking, management, leadership and professionalism. This study explored multiple dimensions of undergraduate dental education in the Middle East and Arabic-speaking African countries and evaluated the curriculum design, teaching and learning methods, assessments, quality assurance and admission criteria in the target institutions.

Responses were received from dental institutions in the Middle East including Saudi Arabia, United Arab Emirates, Kuwait, Jordan, Iraq, Lebanon, and Qatar as well as Arabic-speaking African countries including Egypt, Algeria, Tunisia, and Sudan. Only a small percentage of responses to the survey were provided by the Deans (7.1%) and the majority of responses were provided by their nominated dental faculty representatives who are members of the curriculum committee. Overall, the curriculum structure; teaching and learning methods; assessments; and quality assurance processes are comparable to global trends in undergraduate dental education

albeit with some deficiencies in a small percentage of participating institutions. Given that majority of the institutions have English as the official medium of instruction, along with French, it enables the graduates from these institutions to pursue postgraduate qualifications and License examinations in English/French speaking countries in the United States, Canada, United Kingdom, Australia and some European countries without facing any language barriers.(Bissell et al., 2016)

The admission criteria for undergraduate dental programs are largely similar to those reported from other countries globally. However, candidate interviews were only reported to be a requirement in 42% of institutions. Interviews are a desirable component of admission criteria in many Western countries and may allow a more objective evaluation of communication skills, motivation, readiness for the profession, service, and problem-solving abilities of the prospective candidates. (Duff et al., 2020; Glazer et al., 2016)Nevertheless, it is recognized that conducting interviews of prospective candidates requires a considerable resource, especially for institutions with a large intake and not all dental institutions may be able to reserve such resources routinely. There is growing evidence to support the use of practical tests for admissions to undergraduate dental programs. (Abd Alraheam et al., 2022; Arnold et al., 2011; Beier et al., 2010; Kothe et al., 2014) However, as observed in the present study, only few institutions use practical tests for dental admissions. There is a need to standardize practical tests as a filter for dental admissions in the light of the best evidence and it is conceivable that the trend to utilize practical tests for dental admissions may grow further in the future.

The teaching and learning practices reported by the participating institutions are largely similar to what has been reported from dental institutions globally.(Ali et al., 2016a) Nevertheless, the curriculum development is a dynamic process and needs a continuous review to meet the oral health needs of the communities in the 21st century.(McHarg & Kay, 2008; Perry et al., 2017) A fundamental goal of undergraduate dental education should be to bridge the gulf between basic sciences and clinical disciplines through a meaningful vertical integration to achieve transfer of basic sciences knowledge into clinical practice.(Ali et al., 2020) Technological developments are happening at a lightning pace and the growing use of digital dentistry, immersive realities, and artificial intelligence warrant a careful rethinking of how dental education should be adapted to prepare the dental graduates for the next 40 years. (Ali et al., 2024; Ba-Hattab et al., 2023; Daud et al., 2023; Kim et al., 2023; Monterubbianesi et al., 2022; Philip et al., 2023).

Significant variations were observed in the requirements for clinical targets ($p \leq 0.001$). Nevertheless, the scope of clinical targets and experiences for undergraduate dental students in the present study appears to fulfill the core requirements of a contemporary dental program.(Ali, Slade, Kay, Zahra, & Tredwin, 2017; Ali, Slade, Kay, Zahra, Chatterjee, et al., 2017; Mat Yudin et al., 2020) Notwithstanding the differences in the numbers of clinical targets, it is clear that competence in these core dental skills would allow the dental students to be adequately prepared for their transition into independent clinical practice.(Ali et al., 2016b)

Variations were also observed in the relative weightings of summative and formative assessments amongst the participating institutions with weightings of summative assessments accounting for 50-80% to make decisions regarding student progression. Although there is no gold-standard for relative weightings of summative and formative assessments, it is important that decisions regarding student progression are based assessments which conform with contemporary standards of assessments including the use of multiple assessment strategies and the use of frequent assessments throughout the academic year rather than limited to end of year assessments.(Lockyer et al., 2017)

The results show that all participating institutions have an existing committee structure to oversee the quality assurance of the undergraduate dental programs. However, some apparent deficiencies in quality assurance were identified. Firstly, the frequency of meetings of the quality assurance committee was reported to be once per year or even more by some institutions, which raises some concerns. Quality assurance is a fundamental tool to ensure that contemporary educational and assessment standards are maintained in an educational program and the graduates are safe to provide healthcare services to the communities. (Busari, 2012; Sjöström et al., 2019)Moreover, robust quality assurance mechanisms also enable the institutions to prepare for international accreditation.(Yoshioka & Nara, 2013) It is suggested that institutions with infrequent meetings of relevant quality assurance committees revisit their existing processes and enhance this crucial aspect of dental education to improve the learning experiences of their students. Similarly, some institutions reported that they do not conduct any student surveys at the end of each academic year.(Langan & Harris, 2023; Rigopoulos, 2022; Williams & Kane, 2008) Students are undoubtedly the biggest stakeholders in an educational program, and their feedback serves to inform the future curriculum development, improvements in teaching and learning practices as well as enhancing the quality and transparency of the assessments. Finally, formal qualifications in medical education have been

shown to enhance the agency of educators in healthcare professions.(Du et al., 2022) Many medical and dental institutions in the West require all teaching faculty to have a formal certification in medical education.(Eitel et al., 2000; Fellow-Smith et al., 2013; Görlitz et al., 2015; Schiekirka-Schwake et al., 2017) It is recommended it should be considered a desirable, if not an essential attribute for all dental faculty members. Quality assurance of dental education requires a multi-pronged strategy to evaluate all dimensions of the curriculum, teaching and learning practices, and assessment methods and the afore-mentioned recommendations only indicate some of measures which may contribute to effective quality assurance.

The dental programme structure, duration and admission criteria in the participating institutions are largely similar to those in the United Kingdom and Europe, and Asia.(Chuenjitwongsa et al., 2018; Council, 2015) However, an increasingly number of dental institutions in the West also require an interview as part of the admission process. 14–16 On the other hand, dental schools in the USA and Canada offer a four-year dental programme but applicants require a bachelor's science degree as part of the admission criteria.(*Dental Education | American Dental Association*, n.d.) The Association for Dental Education in Europe (ADEE) has been working to harmonize undergraduate dental education across Europe and has developed the profile and competencies expected from a European dental graduate. (Cowpe et al., 2010; Field et al., 2017) . The aim is to develop uniform standards of undergraduate dental education and allow free movement of dentists across Europe without the need for additional license examinations. Dental regulatory bodies in the Middle East may follow a similar approach and develop a common framework for dental education. It is recognised that it may not be possible to bridge all variations observed in undergraduate dental education, the initial steps would be to develop consensus on the learning outcomes of undergraduate dental education as achieved by ADEE and develop uniformity in how credit hours for undergraduate dental education are calculated. (Cowpe et al., 2010; Kirnbauer & Ali, 2020) Moreover, the dental institutions in the Middle East can also work together to develop and implement a common framework for quality assurance of their dental education programs. A similar approach could be followed by dental institutions in Africa.

This study has some limitations. Although responses were received from the majority of the institutions invited to participate in the study, only a small fraction of dental programs in the Middle East and Arabic-speaking African countries are represented in the study. Therefore the findings may be best regarded as preliminary and need to be interpreted with a degree of caution. Secondly, the findings are based on closed-ended questions and future research

involving qualitative methods may provide a more in-depth understanding of various dimensions of dental curricula and educational strategies at participating institutions along with associated challenges. Furthermore, longitudinal studies may allow more dynamic insights into the trends in evolution of dental curricula. More importantly, all dimensions of curriculum, teaching and learning methods, and assessments could not be explored in a single survey. Further studies with focused questions on specific elements of curriculum design, learning methods and assessments are required for a more comprehensive evaluation. Mapping and alignment of teaching, learning and assessment methods to programme learning outcomes is a key requirement for programmatic assessment in contemporary healthcare education and it is recommended that future studies explore this dimension.(Heeneman et al., 2021) The assimilation and impact of technology in dental education is another topical issue in contemporary dental education and needs to be explored with focused studies on the incorporation of modern technologies such as virtual reality with haptic feedback, digital dentistry, and artificial intelligence. Nevertheless, this study is a first step in evaluation and harmonization of dental education in Arabic speaking countries across the Middle East and African region and would provide impetus to further collaboration amongst the participating institutions.

5 Conclusion

This study provides a snapshot of undergraduate dental education in the Middle East and Arabic speaking African countries. Although a majority of the institutions follow a student-centered approach in the delivery of dental curricula, some in institutions still follow the traditional teacher-centered which is not consistent with the contemporary strategies in healthcare education. The admission criteria to the dental programmes are based on high school grades albeit with some variations. The duration of dental programmes is five years at most institutions with or without an additional foundation year. Variations were also noted in the assessment weightings, clinical targets and quality assurance procedures. Further collaboration is required to facilitate harmonization of dental curricula in the region, solicit international recognition and to better support their graduates in pursuing postgraduate studies and employment options.

Declarations

Research ethics

Ethics approval for the study was obtained from the institutional research ethics committee. Application number D-H-F-11-Nov dated 20th December 2021). Participation in the study was voluntary and all data was processed anonymously.

Availability of data: The data underlying this article will be shared on reasonable request to the corresponding author.

Declaration of generative AI in scientific writing: Generative AI was not used for any aspect of this study including drafting of the manuscript.

Competing interests: The authors have no conflicts of interest to declare.

Funding: Open access funding for this study was provided by the XXXX National Library.

Authors Contributions: KA conceptualized the study, developed the methodology and drafted the manuscript; SAA, HA, MA, and SA contributed to data collection; all authors reviewed and approved the manuscript.

Acknowledgements: The authors would like to thank the dental faculty who participated in this study.

References

Abd Alraheam, I., Oweis, Y., Al-Asmar, A., Ismail, N. H. I., & Sabra, A. H. A. (2022). Predictability of dental students' performance in clinical courses based on their performance in pre-clinical and academic courses. *European Journal of Dental Education*, *26*(4). https://doi.org/10.1111/eje.12760

Ali, K., Barhom, N., Tamimi, F., & Duggal, M. (2024). ChatGPT—A double-edged sword for healthcare education? Implications for assessments of dental students. *European Journal of Dental Education*, *28*(1). https://doi.org/10.1111/eje.12937

Ali, K., Cockerill, J., Bennett, J. H., Belfield, L., & Tredwin, C. (2020). Transfer of basic science knowledge in a problem-based learning curriculum. *European Journal of Dental Education*, *24*(3). https://doi.org/10.1111/eje.12535

Ali, K., Du, X., & Lundberg, A. (2022). Does problem-based learning facilitate enactment of learner agency in undergraduate dental curricula? AQ study. *European Journal of Dental Education*.

Ali, K., Slade, A., Kay, E. J., Zahra, D., Chatterjee, A., & Tredwin, C. (2017). Application of Rasch analysis in the development and psychometric evaluation of dental undergraduates preparedness assessment scale. *European Journal of Dental Education*, *21*(4). https://doi.org/10.1111/eje.12236

Ali, K., Slade, A., Kay, E., Zahra, D., & Tredwin, C. (2017). Preparedness of undergraduate dental students in the United Kingdom: A national study. *British Dental Journal*, *222*(6). https://doi.org/10.1038/sj.bdj.2017.272

Ali, K., Tredwin, C., Kay, E. J., Slade, A., & Pooler, J. (2014). Preparedness of dental graduates for foundation training: A qualitative study. *British Dental Journal*, *217*(3). https://doi.org/10.1038/sj.bdj.2014.648

Ali, K., Tredwin, C., Kay, E., & Slade, A. (2016a). Stakeholders' Perceptions About a Newly Established Dental School with a Problem-Based, Student-Led, Patient-Centered Curriculum: A Qualitative Study. *Journal of Dental Education*, *80*(3), 291–300.

Ali, K., Tredwin, C., Kay, E., & Slade, A. (2016b). Transition of new dental graduates into practice: A qualitative study. *European Journal of Dental Education*, *20*(2). https://doi.org/10.1111/eje.12143

Alshihri, A. A., Salem, D. M., Alnassar, T. M., Alharbi, N. M., Lynch, C. D., Blum, I. R., Wilson, N. H. F., & Aldossary, M. S. (2021). A nationwide survey assessing the satisfaction of dental colleges graduates with their undergraduate experience in Saudi Arabia. *Journal of Dentistry*, *110*. https://doi.org/10.1016/j.jdent.2021.103685

Arnold, W. H., Gonzalez, P., & Gaengler, P. (2011). The predictive value of criteria for student admission to dentistry. *European Journal of Dental Education*, *15*(4). https://doi.org/10.1111/j.1600-0579.2010.00663.x

Ba-Hattab, R., Helvacioglu-Yigit, D., Anweigi, L., Alhadeethi, T., Raja, M., Atique, S., Daas, H., Glanville, R., Celikten, B., Orhan, K., & Ali, K. (2023). Impact of Virtual Reality Simulation in Endodontics on the Learning Experiences of Undergraduate Dental Students. *Applied Sciences (Switzerland)*, *13*(2). https://doi.org/10.3390/app13020981

Balasubramanian, M., Brennan, D. S., Spencer, A. J., & Short, S. D. (2016). The international migration of dentists: directions for research and policy. *Community Dentistry and Oral Epidemiology*, *44*(4). https://doi.org/10.1111/cdoe.12223

Balasubramanian, M., Brennan, D. S., Spencer, A. J., Watkins, K., & Short, S. D. (2015). The importance of workforce surveillance, research evidence and political advocacy in the context of international migration of dentists. *British Dental Journal*, *218*(6). https://doi.org/10.1038/sj.bdj.2015.195

Balasubramanian, M., & Short, S. D. (2011). Is the concept of ethics misplaced in the migration of Indian trained dentists to Australia? the need for better international co-operation in dentistry. *Indian Journal of Dental Research*, *22*(6). https://doi.org/10.4103/0970-9290.94689

Balasubramanian, M., Spencer, A. J., Short, S. D., Watkins, K., Chrisopoulos, S., & Brennan, D. S. (2017). The life story experience of "migrant dentists" in australia: Potential implications for

health workforce governance and international cooperation. *International Journal of Health Policy and Management*, *6*(6). https://doi.org/10.15171/ijhpm.2016.135

Baqain, Z. H., Alshalan, T. A., Naaman, N., & Faleh, S. (2016). An overview of dental education in the Arab world. *Faculty Dental Journal*, *7*(4). https://doi.org/10.1308/rcsfdj.2016.172

Beier, U. S., Kapferer, I., Ostermann, H., Staudinger, R., & Dumfahrt, H. (2010). Impact of a Novel Dental School Admission Test on Student Performance at Innsbruck Medical University, Austria. *Journal of Dental Education*, *74*(5). https://doi.org/10.1002/j.0022-0337.2010.74.5.tb04900.x

Bissell, V., Chamberlain, S., Davenport, E., Dawson, L., Jenkins, S., & Murphy, R. (2016). The Overseas Registration Examination of the General Dental Council. *British Dental Journal*, *221*(5). https://doi.org/10.1038/sj.bdj.2016.645

Busari, J. O. (2012). Comparative analysis of quality assurance in health care delivery and higher medical education. *Advances in Medical Education and Practice*, *3*. https://doi.org/10.2147/AMEP.S38166

Chuenjitwongsa, S., Oliver, R. G., & Bullock, A. D. (2018). Developing educators of European undergraduate dental students: Towards an agreed curriculum. *European Journal of Dental Education*, *22*(3). https://doi.org/10.1111/eje.12306

Council, G. D. (2015). Preparing for practice. *Dental Team Learning Outcomes for Registration*, 16–17.

Cowpe, J., Plasschaert, A., Harzer, W., Vinkka-Puhakka, H., & Walmsley, A. D. (2010). Profile and competences for the graduating European dentist - update 2009. *European Journal of Dental Education*, *14*(4). https://doi.org/10.1111/j.1600-0579.2009.00609.x

Daud, A., Matoug-Elwerfelli, M., Daas, H., Zahra, D., & Ali, K. (2023). Enhancing learning experiences in pre-clinical restorative dentistry: the impact of virtual reality haptic simulators. *BMC Medical Education*, *23*(1), 948.

Dental Education | American Dental Association. (n.d.). Retrieved 4 July 2024, from https://www.ada.org/en/resources/research/health-policy-institute/dental-education

Du, X., Nomikos, M., Ali, K., Lundberg, A., & Abu-Hijleh, M. (2022). Health educators' professional agency in negotiating their problem-based learning (PBL) facilitator roles: Q study. *Medical Education*. https://doi.org/10.1111/medu.14792

Duff, R. E., Katcher, P. A., Daniels, R. M., & Ramaswamy, V. (2020). The Multiple Mini Interview as a Dental School Admission Tool: Can It Predict Noncognitive Traits Associated with Professional Behaviors? *Journal of Dental Education*, *84*(4). https://doi.org/10.21815/jde.019.184

Eitel, F., Kanz, K. G., & Tesche, A. (2000). Training and certification of teachers and trainers: The professionalization of medical education. *Medical Teacher*, *22*(5). https://doi.org/10.1080/01421590050110812

Fellow-Smith, E., Beveridge, E., Hogben, K., Wilson, G., Lowe, J., Abraham, R., Ingle, D., Bennett, D., & Hernandez, C. (2013). Training the Trainers of Tomorrow Today - driving excellence in medical education. *BMJ Quality Improvement Reports*, *2*(1). https://doi.org/10.1136/bmjquality.u201078.w715

Field, J. C., Cowpe, J. G., & Walmsley, A. D. (2017). The Graduating European Dentist: A New Undergraduate Curriculum Framework. In *European Journal of Dental Education* (Vol. 21). https://doi.org/10.1111/eje.12307

Glazer, G., Startsman, L. F., Bankston, K., Michaels, J., Danek, J. C., & Fair, M. (2016). How many schools adopt interviews during the student admission process across the health professions in the United States of America? *Journal of Educational Evaluation for Health Professions*, *13*. https://doi.org/10.3352/jeehp.2016.13.12

Görlitz, A., Ebert, T., Bauer, D., Grasl, M., Hofer, M., Lammerding-Köppel, M., & Fabry, G. (2015). Core competencies for medical teachers (KLM) – A position paper of the gma committee on personal and organizational development in teaching. *GMS Zeitschrift Fur Medizinische Ausbildung*, *32*(2). https://doi.org/10.3205/zma000965,

Gouda, H., Virtanen, J. I., & El Tantawi, M. (2023). Dental public health education in Egypt: a cross-sectional survey. *BMC Medical Education*, *23*(1), 899.

Hajian, S., Jadidfard, M. P., Yazdani, S., Randall, G., & Khoshnevisan, M. H. (2023). Understanding

why oral health professionals migrate: A qualitative investigation of Iranian dentists who have moved to Canada (Oral health professionals' migration). *Global Health Action*, *16*(1). https://doi.org/10.1080/16549716.2023.2190652

Heeneman, S., de Jong, L. H., Dawson, L. J., Wilkinson, T. J., Ryan, A., Tait, G. R., Rice, N., Torre, D., Freeman, A., & van der Vleuten, C. P. M. (2021). Ottawa 2020 consensus statement for programmatic assessment–1. Agreement on the principles. *Medical Teacher*, *43*(10). https://doi.org/10.1080/0142159X.2021.1957088

Kim, C. S., Samaniego, C. S., Sousa Melo, S. L., Brachvogel, W. A., Baskaran, K., & Rulli, D. (2023). Artificial intelligence (A.I.) in dental curricula: Ethics and responsible integration. *Journal of Dental Education*, *87*(11). https://doi.org/10.1002/jdd.13337

Kirnbauer, B., & Ali, K. (2020). Twenty years after the launch of Bologna Process—What is the status of harmonisation of dental education? *European Journal of Dental Education*, *24*(1). https://doi.org/10.1111/eje.12473

Kothe, C., Hissbach, J., & Hampe, W. (2014). Prediction of practical performance in preclinical laboratory courses - the return of wire bending for admission of dental students in Hamburg. *GMS Zeitschrift Für Medizinische Ausbildung*, *31*(2).

Langan, A. M., & Harris, W. E. (2023). Metrics of student dissatisfaction and disagreement: longitudinal explorations of a national survey instrument. *Higher Education*. https://doi.org/10.1007/s10734-023-01004-0

Lockyer, J., Carraccio, C., Chan, M. K., Hart, D., Smee, S., Touchie, C., Holmboe, E. S., & Frank, J. R. (2017). Core principles of assessment in competency-based medical education. *Medical Teacher*, *39*(6). https://doi.org/10.1080/0142159X.2017.1315082

Mat Yudin, Z., Ali, K., Wan Ahmad, W. M. A., Ahmad, A., Khamis, M. F., Brian Graville Monteiro, N. A., Che Ab. Aziz, Z. A., Saub, R., Rosli, T. I., Alias, A., Abdul Hamid, N. F., & Harun, N. A. (2020). Self-perceived preparedness of undergraduate dental students in dental public universities in Malaysia: A national study. *European Journal of Dental Education*, *24*(1). https://doi.org/10.1111/eje.12480

McHarg, J., & Kay, E. J. (2008). The anatomy of a new dental curriculum. In *British Dental Journal* (Vol. 204, Issue 11). https://doi.org/10.1038/sj.bdj.2008.464

Monterubbianesi, R., Tosco, V., Vitiello, F., Orilisi, G., Fraccastoro, F., Putignano, A., & Orsini, G. (2022). Augmented, Virtual and Mixed Reality in Dentistry: A Narrative Review on the Existing Platforms and Future Challenges. In *Applied Sciences (Switzerland)* (Vol. 12, Issue 2). https://doi.org/10.3390/app12020877

Perry, S., Burrow, M. F., Leung, W. K., & Bridges, S. M. (2017). Simulation and curriculum design: a global survey in dental education. *Australian Dental Journal*, *62*(4). https://doi.org/10.1111/adj.12522

Philip, N., Ali, K., Duggal, M., Daas, H., & Nazzal, H. (2023). Effectiveness and Student Perceptions of Haptic Virtual Reality Simulation Training as an Instructional Tool in Pre-Clinical Paediatric Dentistry: A Pilot Pedagogical Study. *International Journal of Environmental Research and Public Health*, *20*(5). https://doi.org/10.3390/ijerph20054226

Rigopoulos, G. (2022). Assessment and Feedback as Predictors for Student Satisfaction in UK Higher Education. *International Journal of Modern Education and Computer Science*, *14*(5). https://doi.org/10.5815/ijmecs.2022.05.01

Schiekirka-Schwake, S., Anders, S., Von Steinbüchel, N., Becker, J. C., & Raupach, T. (2017). Facilitators of high-quality teaching in medical school: Findings from a nation-wide survey among clinical teachers. *BMC Medical Education*, *17*(1). https://doi.org/10.1186/s12909-017-1000-6

Sjöström, H., Christensen, L., Nystrup, J., & Karle, H. (2019). Quality assurance of medical education: Lessons learned from use and analysis of the WFME global standards. *Medical Teacher*, *41*(6). https://doi.org/10.1080/0142159X.2018.1536259

Tonni, I., Gadbury-Amyot, C. C., Govaerts, M., Cate, O. Ten, Davis, J., Garcia, L. T., & Valachovic, R. W. (2020). Adea-adee shaping the future of dental education iii assessment in competency-based dental education: Ways forward. *Journal of Dental Education*, *84*(1). https://doi.org/10.1002/jdd.12024

Williams, J., & Kane, D. (2008). Exploring the NSS: Assessment and feedback issues. *Higher*

Education Academy.

Yoshioka, T., & Nara, N. (2013). International accreditation of medical school towards quality assurance of medical education. *Clinical Neurology*, *53*(11). https://doi.org/10.5692/clinicalneurol.53.1136

Dental Curricula

ORIGINALITY REPORT

8%

SIMILARITY INDEX

PRIMARY SOURCES

1	qspace.qu.edu.qa<br>Internet	67 words — 1%
2	www.synergydentalgroup.net<br>Internet	32 words — 1%
3	www.mdpi.com<br>Internet	29 words — 1%
4	purehost.bath.ac.uk<br>Internet	27 words — < 1%
5	www.researchsquare.com<br>Internet	22 words — < 1%
6	bmcmededuc.biomedcentral.com<br>Internet	21 words — < 1%
7	www2.mdpi.com<br>Internet	21 words — < 1%
8	discovery.dundee.ac.uk<br>Internet	20 words — < 1%
9	assets.researchsquare.com<br>Internet	19 words — < 1%
10	www.science.gov<br>Internet	

19 words — < 1%

11 Andrea Moglia, Konstantinos Georgiou, Pietro Cerveri, Luca Mainardi, Richard M. Satava, Alfred Cuschieri. "Large language models in healthcare: from a systematic review on medical examinations to a comparative analysis on fundamentals of robotic surgery online test", Artificial Intelligence Review, 2024
Crossref
17 words — < 1%

12 journals.plos.org
Internet
15 words — < 1%

13 repository.uob.edu.ly
Internet
14 words — < 1%

14 extremeneoprene.com
Internet
13 words — < 1%

15 vbn.aau.dk
Internet
13 words — < 1%

16 Jack Novovic, Theresa L. Charrois, M. Ken Cor, Jill J. Hall. "Admissions processes in North American pharmacy schools: To what extent are characteristics of practice success measured?", Currents in Pharmacy Teaching and Learning, 2018
Crossref
11 words — < 1%

17 core.ac.uk
Internet
11 words — < 1%

18 digibug.ugr.es
Internet
11 words — < 1%

19 journals.lww.com

Internet

11 words — < 1%

20 play.google.com
Internet

11 words — < 1%

21 Dario Torre, Neil E. Rice, Anna Ryan, Harold Bok et al. "Ottawa 2020 consensus statements for programmatic assessment – 2. Implementation and practice", Medical Teacher, 2021
Crossref

10 words — < 1%

22 Kamran Ali, E. S. A. Alhaija, Mahwish Raja, Daniel Zahra et al. "Blended learning in undergraduate dental education: a global pilot study", Medical Education Online, 2023
Crossref

10 words — < 1%

23 studentsrepo.um.edu.my
Internet

10 words — < 1%

24 J.H. Bennett, J.A. Beeley, P. Anderson, L. Belfield et al. "A Core Curriculum in the Biological and Biomedical Sciences for Dentistry", European Journal of Dental Education, 2020
Crossref

9 words — < 1%

25 Latha S. Davda, Jennifer E. Gallagher, Stephanie D. Short, Madhan Balasubramanian. "Migrant dentists, health system responses and future challenges: a case study of the United Kingdom and Australia", Journal of Ethnic and Migration Studies, 2023
Crossref

9 words — < 1%

26 Martin C.S. Wong. "The Routledge Handbook of Public Health and the Community", Routledge, 2021
Publications

9 words — < 1%

27	www.nature.com<br>Internet	9 words — < 1%
28	www.ukessays.com<br>Internet	9 words — < 1%

EXCLUDE QUOTES	OFF	EXCLUDE SOURCES	OFF
EXCLUDE BIBLIOGRAPHY	ON	EXCLUDE MATCHES	< 9 WORDS
