# Supplementary material for: Undergraduate dental curricula in Middle Eastern and Arabic-speaking African Nations − A cross-sectional study
Source: Saudi Dent J. 2024 Nov 2;36(12):1681–7. doi: 10.1016/j.sdentj.2024.10.003 (PMC11976074; doi:10.1016/j.sdentj.2024.10.003)
Supplement: Supplementary Data 2 [file mmc2.docx]

**Supplementary File: Participating Institutions**

| 1. Algeria | Université Ferhat Abbas de Sétif (UFAS) Faculté de Médecine |
| --- | --- |
| 1. Algeria | Faculté de Médecine de Tlemcen Dr Benzerdjeb Benaouda |
| 1. Egypt | Faculty of Dentistry Cairo University |
| 1. Egypt | Faculty of Dentistry Alexandria University |
| 1. Iraq | Dentistry college. University of Anbar |
| 1. Iraq | College of Dentistry, University of Babylon |
| 1. Jordan | Faculty of Dentistry, Jordan University of Science and Technology |
| 1. Kuwait | Faculty of Dentistry - Kuwait University |
| 1. Lebanon | Faculty of Dental Medicine. St Joseph University |
| 1. Qatar | College of Dental Medicine Qatar University |
| 1. Saudi Arabia | Buraydah Private colleges |
| 1. Saudi Arabia | King Saud bin Abdulaziz University for Health Sciences Riyadh |
| 1. Saudi Arabia | Faculty of Dentistry at Rass |
| 1. Saudi Arabia | Faculty of Dentistry - Albaha University |
| 1. Saudi Arabia | College of Dentistry King Faisal University |
| 1. Saudi Arabia | Ibn Sina National College for Medical Studies Jeddah |
| 1. Saudi Arabia | College of Dentistry Qassim University Buraydah |
| 1. Saudi Arabia | College of Dentistry Al Jouf University |
| 1. Saudi Arabia | College of Dentistry, Najran University |
| 1. Saudi Arabia | College of Dentistry Taibah University, Medina |
| 1. Saudi Arabia | College of Dentistry University of Ha'il |
| 1. Saudi Arabia | College of Dentistry Jazan University |
| 1. Sudan | Gezira University-Faculty Of Dentistry |
| 1. Sudan | Faculty of Dentistry, University of Khartoum |
| 1. Tunisia | Faculty of dentistry of Monastir |
| 1. UAE | College of Dentistry, Ajman University |
| 1. UAE | College of Dental Sciences RAK Medical and Health Sciences University |
| 1. Yemen | College of Dentistry, UST Yemen |
